# A feasibility study of a randomized controlled trial protocol to assess the impact of an eHealth intervention on the provision of dietary advice in primary care

**DOI:** 10.1186/s40814-022-01168-z

**Published:** 2022-09-14

**Authors:** Katherine Jefferson, Michael Ward, Wei-Hsi Pang, JoAnne Arcand

**Affiliations:** 1 Faculty of Health Sciences, Ontario Tech University, Oshawa, Ontario L1G 0C5 Canada; 2 Department of Family Medicine, Queen’s University, Kingston, Ontario Canada

**Keywords:** Sodium reduction, eHealth interventions, Physician counselling, Dietary advice, Feasibility of randomized controlled trial

## Abstract

**Background:**

Canadian sodium intakes remain high despite population-wide sodium reduction initiatives, highlighting the need for personal action in reducing dietary sodium. eHealth interventions support patients in dietary change and assist clinicians in decision-making and delivering care, including provision of advice. To date, impact of diet-focused eHealth tools, like the Sodium Calculator (SC) dietary screening tool, on clinical outcomes has received minimal examination. This study assessed feasibility of a randomized controlled trial (RCT) protocol to examine the impact of the SC, a physician-focused intervention, on the quality of dietary sodium reduction advice provided by physicians to their patients with hypertension.

**Methods:**

Primary care physicians from community-based primary care clinics were randomized to one of two groups: (1) ‘usual care’ for dietary sodium counselling or (2) dietary sodium counselling using the SC (‘experimental group’). The primary endpoint was protocol feasibility defined by the following outcomes: process (e.g. recruitment, retention, protocol adherence, acceptability of intervention), resources (e.g. needs, impact on workflow), and management (e.g. staff requirements). Outcomes were assessed using direct observation, interviews, and questionnaires with patients, physicians, and clinic staff.

**Results:**

Seven physicians (*n* = 4 in experimental group, *n* = 3 in usual care group) and 65 patients with hypertension (48.5% men, 69.8 ± 10.1 years) successfully participated. The main challenges identified is related to recruitment rate (48% for patients, 20% for physicians) and physician protocol adherence (76%). These improved with minor protocol modifications. There were several areas of protocol success such as no disruption to physician workflow, hiring clinic nurses as research staff, having a physician site lead to support physician recruitment, and a ‘Protocol Prompt Form’ to increase physician protocol adherence. Importantly, there was a high degree of acceptability of the SC intervention among experimental group physicians [*n* = 3 (75%)].

**Conclusions:**

The modified RCT protocol was considered feasible. The identified successes can be leveraged, and the risks can be mitigated, during implementation of a full-scale RCT. Assessment of this RCT protocol is an important step in understanding the effectiveness of diet-focused eHealth tools to supporting physician self-efficacy in assessing, monitoring, and implementing dietary advice in routine clinical practice and supporting patients in effective behaviour change.

## Key messages regarding feasibility

1. What uncertainties existed regarding the feasibility of this physician-focused intervention developed to discuss sodium with their patients?

- This novel, pragmatic research protocol that included a behavioural eHealth dietary intervention was implemented into community-based primary care clinics with limited past research experience. The recruitment rate and acceptability of the intervention and research protocol were uncertain. It was also unclear if the developed protocol could be easily integrated into multiple clinic settings in a timely fashion without disrupting clinic workflow while recruiting adequate participants within the study timeframe.

2) What are the key feasibility findings?

- Challenges included both physician and patient recruitment and physician protocol adherence. These issues were mitigated with the assistance of a physician site lead to support physician recruitment, recruiting physicians in one-on-one, face-to-face meetings, enlisting a clinic nurse to help with recruitment, having patients consecutively complete the study in a shorter timeframe, and utilizing a ‘Protocol Prompt Form’ to enhance physician to increase adherence to study protocol, and physician renumeration.

3) What are the implications of the feasibility findings for the design of the main study?

- The feasibility findings will guide the most efficient and effective implementation of a full-scaled randomized controlled trial protocol to determine if the Sodium Calculator is an effective tool to improve the quality of nutrition care delivered by physicians to their patients. By improving the quality of discussions and counselling related to dietary sodium among patients and their healthcare providers, it is hoped that the Sodium Calculator will facilitate reduced sodium intakes and lead to improved health outcomes related to excess dietary sodium.

## Background

Excess dietary sodium is a causal risk factor for high blood pressure, increasing risk for hypertension, cardiovascular diseases, and stroke [1–5]. In 2017, 3 million deaths worldwide were attributed to high sodium intakes alone [6]. To reduce sodium intakes to the recommended level of less than 2300 mg/day [7], population-wide sodium reduction strategies have been implemented globally and typically focus on food reformulation, nutrition labelling policies, and education [8]. Canada’s Sodium Reduction Strategy (2010) includes these elements, but its voluntary approach has not effectively yielded meaningful reductions to the sodium content of Canadian foods, nor to Canadian sodium intakes [7, 9, 10]. The limited impact of these policies highlights the importance of personal knowledge, decisions, and action by individuals in reducing dietary sodium so that the health benefits of sodium reduction can be achieved.

One way to engage individuals in reducing dietary sodium is through the healthcare system. The World Health Organization emphasizes the role of primary care in behavioural counselling to engage individuals in dietary modification (among other risk factors) to prevent and manage chronic disease [11]. This is highly relevant in relation to dietary sodium, since patients are more likely to engage in reducing sodium intake if their healthcare provider recommends it [12]. However, healthcare provider engagement in providing dietary advice in primary care settings may be limited. Studies show the average diet-related discussion between patients and healthcare providers is < 1 min [13], and only ~20% of patients who require dietary counselling receive it [14]. These types of discussions are often considered burdensome by physicians who report several barriers such as lack of time, limited sodium knowledge, and low self-efficacy in counselling about diet [14, 15]. In contrast, several facilitators to the provision of dietary advice have also been reported, such as increased nutrition education in medical school, access to dietitians, and use of EMR tools [16].

Electronic health (eHealth) tools may support feasible and effective dietary counselling interventions in routine clinical practice [17]. In particular, eHealth tools that serve as decision support tools and/or monitor patient self-reported data can support risk factor screening, improve clinician awareness and patient-clinician communication, and shared decision-making, symptom management, and patient satisfaction with care [18, 19]. However, there are few clinically focused eHealth tools to support dietary risk factors, including those focused on sodium. The Sodium Calculator (SC) [20] is an evidence-based eHealth tool that rapidly screens (< 5 min) and provides detailed individualized feedback on dietary sodium [21], including estimates of sodium intake, comparisons to recommendations, and feedback on the dietary sources of sodium: data that is not available in a timely manner using traditional dietary sodium assessment methods [22]. In a proof-of-concept study, the SC improved user sodium knowledge, attitudes, and intended sodium reduction behaviours [23]. Therefore, we hypothesize that the SC, as a physician-focused intervention, can be an effective clinical support tool to support patient monitoring and the delivery of higher quality behavioural counselling for dietary sodium reduction in a clinical setting. We developed a randomized controlled trial (RCT) protocol to evaluate the impact of the SC as an eHealth intervention compared to usual care on the quality of physician-delivered dietary advice related to sodium reduction among patients with hypertension in primary care. The original aim of this study was to implement a full-scale RCT to measure the effectiveness of the SC; however, unanticipated challenges arose during protocol implementation, highlighting the need for a pilot study to determine feasibility. Thus, the objective of the present study was to identify challenges and successes related to protocol implementation and feasibility of the SC intervention, as defined by metrics and observations related to process outcomes (e.g. recruitment rate, retention and challenges, protocol adherence, and acceptability of intervention), resource outcomes (e.g. needs, impact on clinic workflow), and management (e.g. staff requirements, comprehension of protocols).

## Methodology

### Study design

**Fig. 1:**
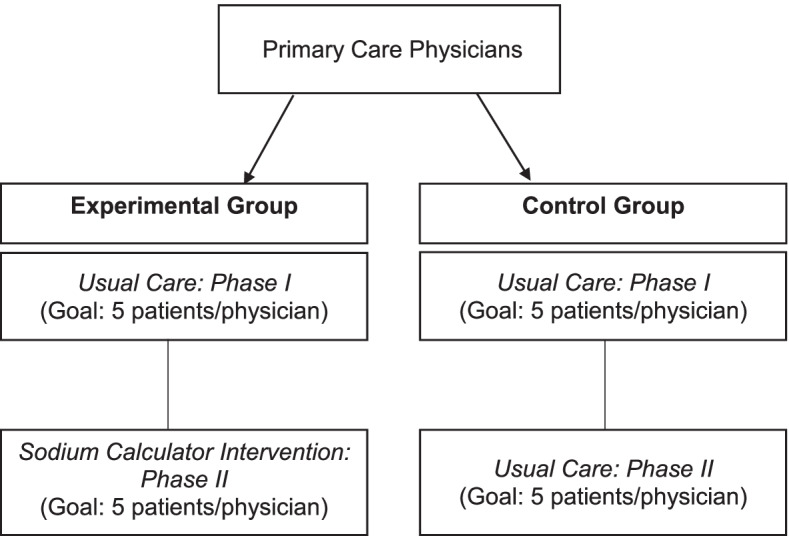
Study design

This study was a pilot study to determine feasibility of a single-blinded parallel randomized controlled trial (RCT) (Fig. 1). Primary care physicians first delivered their usual care for dietary sodium reduction advice to five patients (phase 1, both groups) and then were randomized to continue usual care or to use a novel SC intervention for sodium reduction advice (phase 2). This design allowed for within-physician changes in sodium counselling behaviours to be captured. Interested primary care physicians from four clinics and their patients with hypertension were enrolled. All patients received dietary sodium advice from their physician; however, the method of delivering dietary sodium advice varied depending on physician randomization (Fig. 1).

The primary outcomes of interest were feasibility metrics related to protocol implementation. Iterative changes to the protocol were made throughout study implementation to optimize the protocol and mitigate challenges. The original protocol outcomes were patient-reported quality of sodium advice (frequency, type, and length) and physician confidence in providing this advice. However, these outcomes are not presented in the current study due to the small sample size. Rather, this study assesses the feasibility of administration of the data collection tools to assess these original outcomes. This study occurred between May 2018 to June 2019; however, measurement of feasibility outcomes was initiated when focus shifted to conducting a feasibility study in December 2018. The original protocol, and amendments, was approved by the Ontario Tech University Research Ethics Board (no. 14625).

### Study interventions

Physicians randomized to the SC intervention (experimental) group used the SC with their patients to support dietary counselling. The SC intervention in this study was physician focused and served to guide dietary sodium advice with patients. The SC is a 23-question dietary screening tool that provided the patient and the physician on the estimated average amount of sodium, how this intake compares to dietary sodium recommendations, and sources of dietary sodium consumed. Patients completed the SC in the waiting room on a tablet prior to seeing the physician, with the results converted to a PDF and uploaded to the EMR. Physicians were instructed to review the SC results during the appointment with the patient and then discuss dietary sodium reduction using their clinical judgement. Thus, discussions about dietary sodium were physician led and tailored to the patient and varied between patients. *Usual care* in this study was defined as the current practices of each physician for sodium reduction with their patients. Physicians followed their usual schedules and clinical practices, with the exception that they were required to discuss dietary sodium reduction in the appointment.

### Inclusion/exclusion criteria

Physicians were eligible if they worked in one of the participating primary care clinics, provided care to patients with hypertension, and were fluent in English. Patients were eligible if they had a scheduled appointment related to blood pressure management or an annual/biannual health exam and were over 18 years of age with a new or existing diagnosis of hypertension (seated resting blood pressure in clinic of ≥ 140/90 mmHg) [24] with either controlled or uncontrolled blood pressure with or without the use of anti-hypertensive medication. Excluded were patients with a diagnosis of dementia, history of an event affecting memory (memory was required to complete the SAQ), or with visual impairments limiting their use of an electronic tablet device to complete the SC.

### Measurement of study outcomes

The outcomes focused on protocol feasibility, organized by relevant process, resource, and management indicators [25]. The most pertinent outcomes are presented in this paper: physician and patient recruitment rate, adherence rate, and physician acceptability of the intervention. Most feasibility outcome measures were collected throughout the entirety of the study (13 months), based on direct observations by study personnel and discussions with patients, physicians, and clinic staff, and collected from tallies and demographic and acceptability questionnaires. However, some feasibility outcome measures, such as recruitment rate, were collected; once it was determined, a feasibility study was required, and the full-scale RCT was halted in December 2018. As part of this protocol, all data collection procedures as intended with the RCT were conducted. This included administration of questionnaires to capture the frequency and duration of patient-reported dietary sodium advice from physicians and physician self-efficacy in providing this advice. Sodium advice was assessed by a patient-reported 11-item (multiple choice) Sodium Advice Questionnaire (SAQ) completed immediately after seeing the physician. The SAQ was developed and validated for face and content validity by our team for this study protocol. It was designed to be administered post-appointment as it assesses sodium-related topics discussed in the physician-patient interaction. Specifically, questions assess if sodium was discussed, what was discussed, and for how long. Adherence to the study protocol was defined as the provision of dietary sodium advice to patients, which was assessed using the SAQ in a dichotomous manner (completed? yes/no) in both experimental and usual care. Physician self-efficacy in providing sodium reduction advice, as assessed at the end of the study period, was via physician self-report using an online 14-item (Likert scales) Physician Self-efficacy in Sodium Counselling (PSSC) scale questionnaire. This scale was adapted from a validated scale to assess physician self-reported confidence in obesity counselling [26] to assess sodium counselling specifically for this study. Data on these endpoints have not been analysed and reported due to a small sample size and emphasis being on feasibility endpoints.

### Sample size justification

As this is a feasibility study, no sample size was calculated; data was collected data as per our time and resources. Our sample size of seven physicians and 65 patients was felt to be sufficient for the assessment of protocol implementation and feasibility endpoints related to recruitment rate and retention, protocol adherence, and acceptability among physicians and patients across 4 primary clinics. The sample size for a future full-scale RCT trial will be determined based on data collected in this study and will include an effect size with 80% power.

### Recruitment

Primary care physicians were recruited from multiple sites in commuter communities within the Greater Toronto Area in Ontario, Canada. These clinics service both urban and rural residents who are mostly Caucasian with an average age of 38.9 years and median annual total income of CAD $41,820 [27]. Each clinic varied in size, ranging from four to fourteen physicians. These clinics all had limited experience participating in research studies (e.g. recruitment, protocol implementation). Patient caseloads varied per physician, and physician groups included a mixture of full-time and part-time employment. One physician at each clinic became the designated site lead for the project and provided guidance and support related to recruitment. Physicians were recruited via email invitations from the site lead at three clinics. However, at one clinic, a research group information session was given during a lunch rounds presentation. Patient recruitment occurred consecutively through weekly review of physician schedules and patient clinical chart reviews and were contacted by phone to determine interest in participating. These procedures were conducted by a clinic nurse hired for additional hours at each site to serve as a designated research nurse on top of their clinic duties.

### Study protocol

**Fig. 2:**
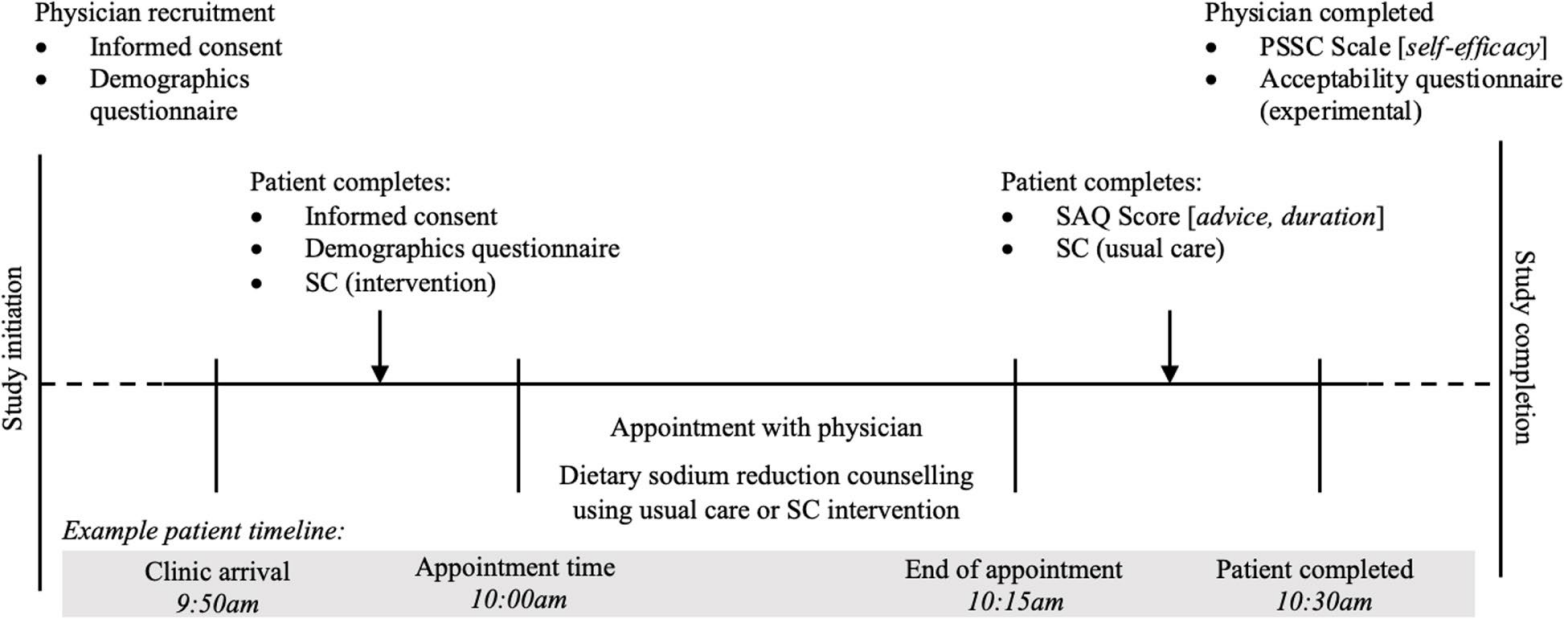
Study protocol overview

Physicians were randomized in a 1:1 ratio into either the experimental group or control group based on computer-generated ID number. To minimize bias and risk of co-intervention, physicians were blinded to the true study objectives and were told that the study was to assess patient-focused outcomes of sodium knowledge, attitudes, and behaviours. Among physicians randomized to the experimental group, study personnel provided a brief orientation to the SC, including two low-sodium diet information pamphlets to support the physician’s use of the SC. Physician training for the SC intervention included the rationale for the SC, where to find the results of the SC, and a review of outputs/results. Physicians in the experimental group provided usual care sodium reduction advice to the first five recruited patients without support of the SC data, followed by five patients where they had access to the SC results. Patients of participating physicians came to their appointment early to complete consent, a demographic questionnaire, and the SC if they were a patient of a physician randomized to the experimental group. When a study patient was visiting a physician in this group, a physician was informed via an EMR message that served as a reminder to implement the study protocol. There were no patients completing the study in the waiting room at the same time, reducing the risk of contamination. In contrast, physicians in the control group provided usual care for all patients. All patients receiving usual care completed the SC after their appointment so that their sodium intake could be estimated but minimize contamination of the appointment. On completion of the appointment, the patient met with study personnel again to complete the SAQ based on their discussion with the physician. All patients completed the SAQ immediately after their visit with their physician. If a physician did not discuss dietary sodium with a study patient, the patient was unable to complete the SAQ, and a new patient was recruited in their place so that each physician would successfully implement the study protocol among 10 patients. Patients could only participate in the study once. Upon study completion, physicians completed the PSSC scale, as well as an acceptability questionnaire to assess the study protocol. Physicians were given a CAD $50 gift card, and patients were given a CAD $10 gift card for participating in the study (Fig. 2).

### Data analysis

Data analysis included descriptive statistics. Continuous data is described as means and standard deviations and categorical data as frequencies and percentages. Likert scale responses from the SC acceptability questionnaire were collapsed into the following categories: ‘strongly agree’ and ‘agree’ into an ‘agree’ category and ‘strongly disagree’ and ‘disagree’ into a ‘disagree’ category. SPSS version 25.0 was used [28]. A comprehensive assessment of data was conducted in consideration of the criteria to assess feasibility provided by Thabane’s framework [25]: (1) stop — main study not feasible, (2) continue but modify approach — feasible with modifications, (3) continue without modifications but monitor closely — feasible with close monitoring, and (4) continue without modification — feasible as is. The ultimate decision of classification of overall feasibility of the study protocol was determined from a pragmatic point of view based on the feasibility of the protocol on recruitment, data collection, and a minimal impact on clinic workflow and based on the judgement of the research team.

## Results

Physicians in the control group (*n* = 3) were older (51.3 ± 2.6 versus 42.8 ± 4.8 years old) and had been practicing for longer (20.0 ± 2.9 versus 14.3 ± 4.0 years) compared to those in the experimental group (*n* = 4) (Table 1). All physicians agreed that health was greatly affected by diet, and most thought it would be helpful if their EMR included nutrition decision support tools (86%). Eighty-six patients were recruited over the entire duration of the study (13 months); however, only 65 patients were included. Twenty-one patients could not be included in the analysis due to missing data; when physicians did not discuss dietary sodium with the patient, a patient was unable to complete the SAQ. Among patients (*n* = 65), 48.5% were men, predominantly Caucasian, with an average age of 69.8 ± 10.1 years old (Table 1).

**Table 1:** Participant demographics

	**Physicians (*n*= 7)**			
	**Experimental group (*n*= 4)**		**Control group (*n*= 3)**	
Age (years)	42.8 ± 4.8		51.3 ± 2.6	
Gender, male	3 (75)		2 (67)	
Length of time in practice (years)	14.3 ± 4.0		20.0 ± 2.9	
Past nutrition education				
A few lectures during medical school	4 (100)		3 (100)	
Workshops or webinars	0 (0)		0 (0)	
A nutrition course	0 (0)		0 (0)	
Postsecondary degree in nutrition	0 (0)		0 (0)	
	**Patients (*n*= 65)**			
	**Experimental group**		**Control group**	
	**Phase 1** **Usual care (*n*= 18)**	**Phase 2** **Sodium Calculator (*n*= 18)**	**Phase 1** **Usual care (*n*= 15)**	**Phase 2** **Usual care (*n*= 14)**
Age (years)	70.4 ± 8.8	69.9 ± 11.0	73.1 ± 10.5	66.2 ± 10.4
Gender, male	8 (44.4)	9 (50.0)	15 (52)	9 (64.3)
Number of antihypertensive medications	1.6 ± 0.5	1.4 ± 0.92	2.1 ± 1.4	1.5 ± 1.0
Systolic blood pressure (mmHg)	137 ± 18.4	141.3 ± 16.2	139 ± 19.1	137 ± 18.3
Diastolic blood pressure (mmHg)	78 ± 13.6	76 ± 10.7	78 ± 14.5	82 ± 12.9
BMI (kg/m^2^)	34.6 ± 6.6	30.0 ± 6.3	31.1 ± 8.0	32.3 ± 4.2
Received advice from a dietitian about dietary sodium in the past	2 (11.1)	0 (0.0)	2 (13.3)	2 (14.3)
Received advice from physician about dietary sodium in the past	6 (33.3)	3 (16.7)	5 (33.3)	6 (42.9)
Sodium/salt affects your blood pressure				
°Agree	17 (94.4)	15 (83.4)	12 (80.0)	10 (71.4)
°Neutral	1 (5.6)	2 (11.1)	1 (6.7)	3 (21.4)
°Disagree	0 (0.0)	0 (0.0)	2 (13.3)	1 (7.1)
°N/A	0 (0.0)	1 (5.6)	0 (0.0)	0 (0.0)
Currently trying to follow a low-sodium diet (pre-intervention)	7 (38.9)	9 (50.0)	5 (33.3)	5 (35.7)

Continuous data are presented as means ± standard deviation. Categorical data presented as frequency (percent)

### Process outcomes

#### Recruitment rate: Physicians (Fig. 3) — email recruitment

Among three clinics, twenty-one primary care physicians received an email invitation to participate in the study from the site lead (physician). Ten physicians stated interest in participation, and nine provided informed consent (9/21 physicians, 43% recruitment rate via email method).

**Fig. 3:**
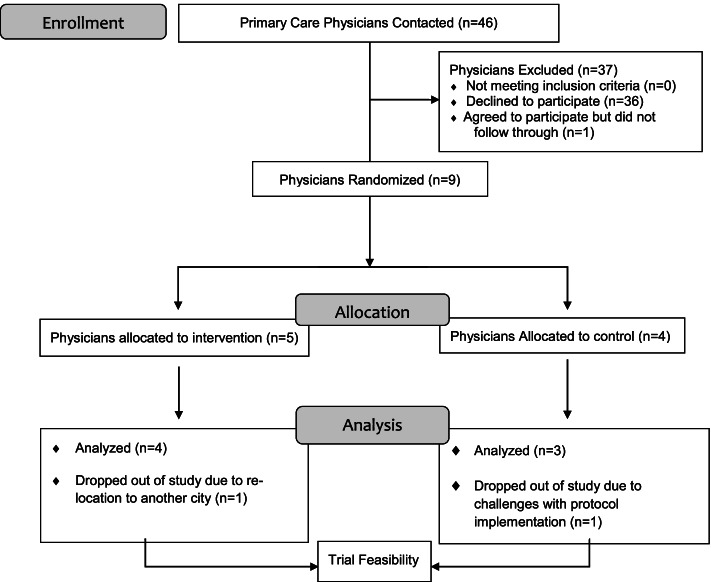
Participant flow: physicians

#### Physicians —Information session recruitment

At one clinic, twenty-five physicians were invited to a study information session. Seven physicians attended the session, and none agreed to participate (0/25, 0% recruitment rate via information sessions), with lack of time and being new to practice being the stated reasons for nonparticipation. The overall physician recruitment rate was 20% (9/46 physicians contacted). Feedback from site leads indicated that interest in participation would increase if physicians were renumerated more for time spent on research.

#### Patient recruitment (Fig. 4)

Recruitment rate was calculated based on the available data from December 2018 onwards. A total of 1602 patients were screened, 387 had hypertension (24%), and 151 of these 387 patients met the full eligibility criteria (39%). Study personnel called the 151 patients by phone. Overall, 106 (80.2%) patients were successfully contacted, and 51 provided informed consent (51/106 = 48% recruitment rate).

**Fig. 4:**
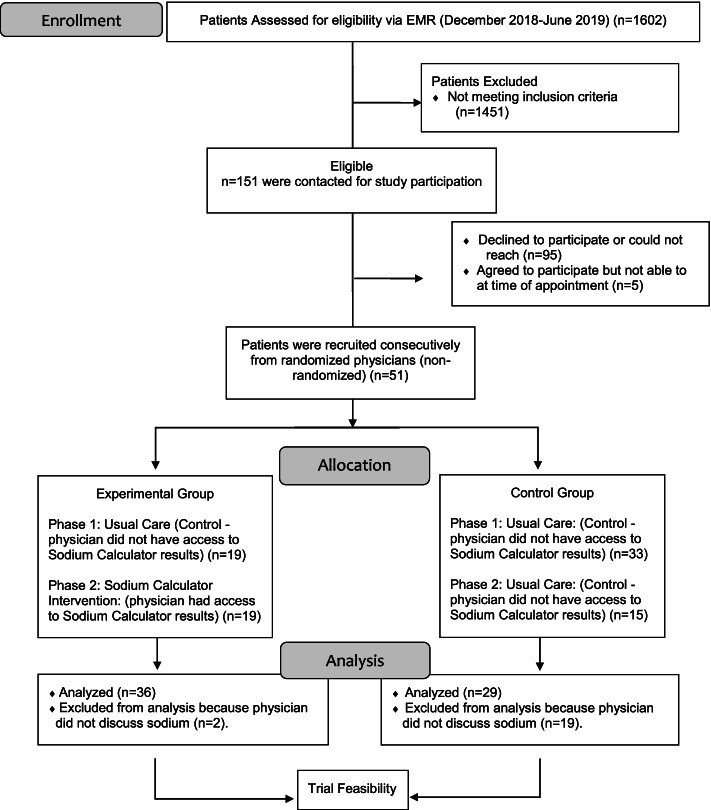
Participant flow: patients

#### Challenges with patient recruitment

Study personnel noted that it was difficult contacting patients by telephone. For all patients contacted, the hired clinic nurse assessed the common reasons for patients declining participation, which were a lack of time (especially those employed, < 65 years of age), logistical challenges with appointments (e.g. reliance on someone else for transportation), disinterest in research participation, lack of concern about blood pressure, and the desire for maximal time for their doctor to focus on other medical priorities. Patient eligibility criteria were initially found to be restrictive and became a significant barrier to recruitment. The principal limiting criterion was the requirement that patients had to have been scheduled for an annual health exam or blood pressure follow-up. This criterion was chosen since these types of appointments are when dietary sodium reduction counselling is most likely to occur. In an attempt to improve recruitment rate, mid-way through the study, the eligibility criteria were broadened to include patients who had pre-hypertension or three or more risk factors for developing hypertension. However, this did not result in a substantive increase in the recruitment rate as this information was not always readily available in patient charts at these clinics when screening.

#### Participant retention

One physician in the experimental group dropped out of the study due to relocation to another country. One physician in the control group dropped out of the study due to challenges with protocol implementation, as they worked part time in their clinic, did not have many eligible patients, and consistently forgot to implement the study protocol (e.g. only 1 patient had complete data over a 12-month period). There were no patient dropouts.

#### Adherence to study protocols

The overall physician protocol adherence rate was 76% (65/86 of patient appointments), as defined by discussing dietary sodium with their patients measured by the SAQ; however, the rate of adherence increased over time from 42% (7/12 patient visits) in the first 3 months to 87% (1/27 patient visits) by the last 3 months of the study. Approximately, half of the physician non-adherence cases are attributed to one physician in the control group who eventually dropped out of the study. The majority of non-adherence occurred in the first 5 patients (usual care) (control group *n* = 14 patients; experimental group: *n* = 5 patients). Phase 2 had significantly fewer patients unable to complete the study with *n* = 1 patients of physicians in the experimental group and *n* = 1 patients of physicians in the control group.

Failing to discuss sodium with the patient was the cause for most instances of protocol non-adherence, which occurred despite reminders and colour coding (flagging) study patients in their electronic chart. Data from twenty-one recruited patients overall (24%) were unable to be included in the study as their physician was not adherent to the protocol (e.g. patients had missing data because the SAQ could not be completed). When a physician failed to discuss sodium with a patient participant, additional patients were recruited so that the physician could aim to successfully implement the study protocol for the required number of patients. The most common reason for physicians failing to discuss sodium with patients was forgetting to discuss sodium, followed by not having enough time or having more pressing matters to discuss with the patient. As an attempt to increase protocol adherence, a ‘physician protocol prompt form’ was introduced, a brightly coloured hard copy form that notified the physician that the patient was participating, and they were required to discuss dietary sodium (in both experimental and control groups). The benefits of the form were noted by physicians at the end of the study: ‘The patient having a physical piece of paper was a very good reminder that I had to review the sodium calculator results’ — Physician 1. Study personnel also observed that scheduling patients more consecutively (i.e. fewer sporadic study participant visits) also enhanced physician implementation of the protocol, which subsequently accelerated a physicians’ time to study completion. Physicians and patients that successfully completed the study had no difficulty completing the questionnaires, and there was no missing data. For the SC acceptability questionnaire, physicians had the option to provide qualitative responses to elaborate on the acceptability of the tool if desired. Only 2/4 physicians provided qualitative insights.

#### Physician acceptability of the SC intervention

Four physicians in the experimental group completed an SC intervention acceptability questionnaire. Most [*n* = 3 (75%)] thought the SC was useful and provided a better estimate of sodium intake than what they could discern from a brief conversation with their patient. “I think it was useful. Definitely a better estimate of sodium intake than I can come up with after a brief conversation” — Physician 2. There was high agreement that the SC allowed them to get the most out of their time with their patient [*n* = 3 (75%)], and 2/4 physicians (50%) disagreed that the SC took too much time with their patients, while the other 2/4 were neutral. Integrating the SC into clinical practice was thought to be a good idea [*n* = 3 (75%)], and that it easily integrated into their busy workflow [*n* = 3 (75%)]. Of note, 2/4 (50%) disagreed that sufficient training was provided on use of the SC.

### Resource outcomes

#### Impact of research on clinic workflow

Despite some physicians’ concerns prior to enrollment, there was no evidence that the study protocol significantly impacted clinic workflow as evidenced by no delay in the physicians’ appointment schedule during the study period caused by the protocol. Study personnel included a hired clinic nurse who worked at the clinic. They were trained to screen and contact eligible patients for research participation. Observed benefits of having a nurse hired at each clinic were familiarity with the patients and the ability to readily remind physicians about when study patients were scheduled.

#### Time commitment: Physicians

There was no evidence that the study protocol significantly impacted the timing of patient appointments. Physician schedules were not delayed due to the protocol as per verbal confirmation from physicians and the hired clinic nurse assisting with recruitment. No physicians reported concerns with the time commitment of the study protocol, including those that used the SC: “I did not do the calculation with them, just reviewed results so not time consuming” — Physician 4. In the majority of appointments (65%), physicians discussed dietary sodium in 1 to 4 min (patient reported).

#### Time commitment: Patients

Overall, it took patients in both study groups 15–45 min (average 30 min) to complete consent, demographic data collection form, and the SC, see their physician, and complete the SAQ. All patients felt the time required for study participation was reasonable.

### Management outcomes

Clinic appointments were scheduled in 10- or 15-min time slots. It was observed that multiple research assistants to support consent and to complete data collection, before and after the clinic appointment, helped to prevent disruption to a physician’s schedule and clinic flow. The average length of time for physicians to be onboarded to the study, conduct the intervention with 10 patient participants, and complete the acceptability questionnaire was 5.1 months. Two physicians had significant delays due to scheduled vacations or a lack of identified eligible patients. Some patients required guidance and support in using the mobile tablet device, which was largely due to inaccessible reading glasses.

### Overall facilitators and barriers to implementation of the protocol

Overall, there were a number of factors and methodologies that were found to support and impede successful implementation of the original study protocol. These are important considerations for the development of a large-scale cluster randomized controlled trial and are described below (Table 2).

**Table 2:** Key factors for successful protocol implementation

**Physician recruitment**	
• Engagement of a site lead investigator (physician) to assist with physician recruitment and protocol implementation — via email is encouraged	
• One-on-one, face-to-face meetings with physicians, rather than group information sessions	
• Offer sufficient renumeration to offset concerns and income lost from time required for research. One participant recommended a minimum of 150 Canadian dollars	
**Patient recruitment**	
• Enlisting a nurse at each clinic to assist the research team with patient recruitment. Ensure buy-in of this strategy from physicians at clinic prior to implementing	
• Consider liberalized eligibility criteria that will allow the maximum number of patients to participate	
**Protocol adherence**	
• Having physicians see study patients more consecutively over a shorter period of time	
• Sending study patients into the clinic room with a visible, tangible hard copy of a ‘physician prompt form’, with additional reminders for physicians from study personnel for the first few patients that complete the study	

### Overall assessment of study protocol feasibility

Based on the data generated, the research protocol was not initially deemed feasible; however, the modifications made to protocol adherence strategies demonstrated improvements and increased the rate of successful patient recruitment and completion rate. In the experimental group, the SC intervention was positively viewed and considered to have potential for integration into clinics by three of the four physicians. Overall, the original strategy of having a physician site lead at each clinic, hiring clinic nurses to assist with patient recruitment, and increasing the number of research assistants in clinic to assist with patient intakes allowed for minimal disruption on clinic workflow. Therefore, it is concluded that this updated protocol is appropriate to be successfully scaled up to a large multicentre RCT.

## Discussion

In this in-depth assessment of study protocol feasibility, this modified RCT protocol was successfully implemented in multiple busy primary care clinics with varying staff and administrative procedures. This study has produced insights into the feasibility of conducting a physician-focused RCT to assess the impact of a dietary assessment tool on the quality of physician sodium reduction advice. The realization that a feasibility study was needed became apparent when challenges with implementing the RCT study protocol were experienced, and through the process of collecting data on feasibility endpoints, potential solutions were determined. In part, challenges experienced early on in this protocol may be related to broader challenges in the delivery of dietary advice in primary care practice [16]. Regardless, the findings in this study will increase success of the full-scale RCT and may support other nutrition researchers in designing and implementing similar studies. Additionally, although only a small number of physicians experienced using the SC as a tool to aide their dietary sodium counselling, overall, there was positive response to the tool and integrating into practice. This warrants further examination of the SC and its effectiveness on supporting the delivery of dietary sodium reduction advice in clinical settings and justifies the development and evaluation of other similar nutrition-focused eHealth tools and interventions.

There are several successes and challenges of this protocol that warrant discussion. Recruitment is a commonly documented challenge in RCTs [29, 30]. In this study, a dedicated site lead at each clinic location to champion the research was a critically important factor in maximizing physician recruitment and minimizing research impact on clinical workflow. In contrast, group research information sessions with physicians were an ineffective recruitment approach. One challenge impacting physician recruitment may have been the community-based nature of the primary care clinics included, which had minimal past research exposure. Indeed, lack of research experience and an organizational culture that values research endeavours have been linked to greater unwillingness of physicians to participate in research studies [31–34]. However, it is critical to conduct research in these settings to produce generalizable data outside of clinical academic environments, where most trials are conducted. Although the site lead was a key facilitator in recruitment, a future recommendation to enhance physician recruitment in a scaled-up RCT is to partner with a physician research network to increase recruitment efficiency and reach, a strategy known to yield an unbiased sample of physicians [29, 35]. Additionally, allowing physicians in participating clinics to inform the study protocol as part of an integrated knowledge translation approach would enhance feasibility, acceptability, and overall implementation of the research [36], as has been found in settings with participants with little research experience as part of community-based participatory research [37]. Finally, physicians who did not consent to participate voiced concerns about a lack of time to participate in research, another documented factor that limits research participation [38]. However, our findings indicate that the majority of physicians felt the study protocol did not impact workflow and took a reasonable amount of time to implement. These findings occurred in addition to documented benefits of providing advice using the SC including increased ease, accuracy, and support of providing advice about dietary sodium.

A slow rate of patient recruitment is commonly reported in RCTs, with only a minority of trials successfully recruiting a planned sample size within the anticipated recruitment timeframe [30]. Although hypertension is common in Canada, with a prevalence of 23% overall [39], this study experienced challenges in patient recruitment. This study included only patients with an upcoming appointment for a blood pressure check-up or annual/biannual health exam, which was found to be the most significant limiting factor to patient recruitment. However, taking this approach enabled us to pragmatically test the SC intervention as part of a realistic clinical scenario, since discussion about dietary sodium is most likely to occur during these types of patient-physician interactions. Broadening patient eligibility criteria to include patients with risk factors for hypertension did not result in a substantial increase in patient recruitment as it was difficult to determine patients with multiple hypertension risk factors through clinical chart screening. A key recommendation based on the data generated in this study is to identify and recruit patients through a review of a physicians’ patient roster and then to schedule interested, eligible patients for their blood pressure follow-up over one or two pre-arranged clinic days. This approach would expand patient eligibility criteria by removing the type of appointment. This may also help increase recruitment efficiency and also physician adherence to protocols.

Issues with physician adherence to study protocols were evident in this study, particularly in the early stages. However, initial issues with physician adherence were largely mitigated, resulting in an overall adherence of 76% of patient appointments with increases seen with the use of a ‘physician protocol prompt’ form and when study patients were scheduled more consecutively. As the majority of non-adherence occurred in *phase 1* (usual care), this may signify that there is a period where physicians are becoming accustomed to seeing study patients, and that their adherence to protocol improves with the more study patients seen. Therefore, more support and reminders from study personnel would be beneficial early on in protocol implementation for each participating physician. In other research, protocols with more extensive physician-focused interventions have found difficulties in physician protocol adherence as well [40], although the literature has tended to focus on patient, rather than physician non-adherence.

Although it did not impact the results of this feasibility study, there were differences noted in both physician and patient demographic data between the groups that deserves note. Some research has shown that older physicians > 50 years have higher rates of assessing patient dietary habits, and female physicians are more likely to follow the 5As (Assess, Advise, Agree, Assist, Arrange) counselling framework [41]. In the small sample size of this feasibility study, we recruited more male physicians and had a large difference in mean age and practice (8.5 years and 5.7 years, respectively), which based on minimal existing literature may suggest an impact the sodium advice physicians give their patients, which could confound the efficacy results of the SAQ score between the experimental and control groups. Patient characteristics such as age, ethnic background, and type and number of chronic disease diagnoses also have been shown to impact provision of dietary counselling, which may have impacted physician protocol adherence in this study [13, 42, 43]. Possible confounding factors related to physician provision of counselling should be accounted for in the RCT study design, with stratification of these demographics recommended.

The majority of physicians supported the benefits of the SC when used as a part of this pragmatic study protocol, which is in line with previous findings that tailored eHealth technologies are well accepted or regarded by physicians, as they can be helpful in detecting, assessing, and managing patient symptoms and can save time [44, 45]. eHealth interventions have also been shown to improve many components of optimal care as they can improve communication between healthcare provider and patient, provide more patient-centered care, reduce the gap of provision of care, and show clinical management improvement and improved diagnoses [46–51]. The ability of tailored eHealth tools to minimally impact practice has also been found to be appreciated, as was their ability to provide real-time synthesis and analysis of patient data. eHealth tools also have the ability to remind physicians to counsel and provide linked resources to facilitate structured, evidence-based approaches to counselling [52, 53]. Use of an eHealth intervention in cardiovascular disease care has demonstrated increased healthcare provider self-efficacy, improved workflow, and appropriate management of patients [49]. This highlights the importance of continuing this work with the SC as a large-scale RCT while showing evidence of the potential of the SC to improve patient care.

There were strengths and limitations to this study. We collected feasibility outcome data from four different primary care clinics, with varying staff and procedures. The clinics included in this study were in the same geographic region, and a future trial would ideally have increased geographic diversity. However, the clinics included in this study capture novel data as they were community based and not academic centres, which increases the generalizability of the findings. Another strength was that self-reported measures were developed or adapted and validated specifically for this study protocol. A future trial would want to consider improving geographic diversity. The protocol was designed to minimize recall and reporting bias by having physicians and patients complete these self-reported measures immediately on completion of the study. Based on the nature of the intervention, it was not possible to blind study personnel to physician group allocation. Additionally, although physicians were not informed of the true study objectives, they were aware that study patients were required to meet with study personnel after their appointment and may have changed their care due to the Hawthorne effect [54]. Finally, contamination and co-intervention may have occurred in this study since individual physicians in the same clinic were randomized to different study groups; ideally, a scaled-up RCT protocol would be implemented as a cluster RCT to minimize this risk.

In conclusion, the modified RCT protocol was considered to be successful, and key factors related to the successes and challenges to protocol implementation were identified. These successes can be leveraged, and the risks can be mitigated with implementation of a full-scale RCT. As was experienced first hand with this research project, feasibility studies prior to the implementation of full-scale RCT’s are imperative to successfully develop and implement RCTs. The development of this RCT protocol was an important step in understanding the effectiveness of the diet-focused eHealth tools to supporting physician self-efficacy in assessing, monitoring, and implementing dietary advice in routine clinical practice, in a nonacademic community primary care setting, and in supporting patients in effective behaviour change.

## Data Availability

The datasets used and/or analysed during the current study are available from the corresponding author on reasonable request.
